# Biology of *Nicotiana glutinosa* L., a newly recorded species from an archaeological excavation site in Egypt

**DOI:** 10.1186/s12870-024-04816-z

**Published:** 2024-02-28

**Authors:** Selim Z. Heneidy, Yassin M. Al-Sodany, Amal M. Fakhry, Sania A. Kamal, Marwa Waseem A. Halmy, Laila M. Bidak, Eman T. El kenany, Soliman M. Toto

**Affiliations:** 1 Department of Botany & Microbiology, Faculty of Science, Alexandria University, P.O. Box 21511, Alexandria, Egypt; 2 Department of Environmental Sciences, Faculty of Science, Alexandria University, P.O. Box 21511, Alexandria, Egypt; 3 Department of Oral Biology, Faculty of Dentistry, Pharos University in Alexandria, Alexandria, Egypt

**Keywords:** *Nicotiana glutinosa*, Solanaceae, New record, Size structure, Egypt, Invasive

## Abstract

**Background:**

During a field survey of urban flora in Alexandria city in 2019–2022, an interesting species belonging to the Solanaceae was collected from a newly archaeological excavation site and identified as *Nicotiana glutinosa* L. Many visits were made to the herbaria of Egypt to confirm the species records, but no single record was found. Reviewing the available literature revealed that this tropical American taxon was never recorded in the flora of Egypt.

**Aims:**

The present study was focused on *N. glutinosa* growth structure and plant macro- and micromorphology.

**Methods:**

Ten sampling sites were covered for *N. glutinosa* size structure. Plant samples were examined for stem anatomy, leaf, seed, and pollen morphology.

**Results:**

The species size structure reveals that the individual size index ranges from 1.33 to 150 cm, while its density ranges from 4 to 273 individuals /100 m^−2^.

*N. glutinosa* has successfully established itself in one of the archaeological sites in Egypt, showing a “healthy” population with a high degree of size inequality, characterized by a relative majority of the juvenile individuals. Voucher specimens were deposited in the Herbarium of Alexandria University (ALEX) Faculty of Science, another specimen is processed to make herbarium specimens at the Herbarium of the Botanic Garden (Heneidy et al. collection, deposition number. 5502).

**Conclusions:**

From our observations, *N. glutinosa* seems to have invasive potential, as it shows characteristics shared by most invasive species that are thought to help in their successful establishment in new habitats. This article emphasizes the importance of monitoring and regularly reporting the threats of alien invasive species to avoid any possible negative impacts on indigenous biodiversity in the future.

**Supplementary Information:**

The online version contains supplementary material available at 10.1186/s12870-024-04816-z.

## Introduction

Alien invasive species are a major threat to global biodiversity, as they may alter ecosystem processes and functions [1]. Many alien plant species are introduced accidentally across the globe. Some species may become invasive within an innovative ecosystem [2]. Therefore, it is important to understand the mechanisms by which alien invasive species might become serious threats to native biodiversity. During frequent field surveys of the urban flora of Alexandria, Egypt, a new alien species, *Nicotiana glutinosa* L., was recorded in a newly discovered archaeological site.

*N. glutinosa*, an annual herb or short-lived perennial plant species, belongs to Solanaceae. It is a species of tobacco plant that is economically important in tobacco hybrids. The plant is native to western South America, including Bolivia, Ecuador, and Peru. It is a model organism for studying Tobacco Mosaic Virus (TMV) resistance in tobacco [3]. Because the plant has lovely flowers, it can be used as a cut flower [4]. *N. glutinosa* is the source of the Tobacco Mosaic Virus resistance gene (N) bred into commercial tobacco [5]. Potato tuber worms eat this species.

As the increased understanding of the existence of newly recorded species and their habitats can help to detect, evaluate, monitor, and anticipate changes in biodiversity and its conservation, the current study carried out on *N. glutinosa* in Egypt highlights the new record and novel taxon addition to the flora of Egypt. In this article, we aim to identify and describe this species, referring to its growth structure and plant macro- and micromorphology.

## Nomenclature

*Nicotiana glutinosa* L., Sp. Pl. 181. 1753

### Synonyms

Sairanthus glutinosus (L.) G. Don, Gen. Hist. 4: 467 (1838)

*Tabacus viscidus* Moench, Methodus: 448 (1794), nom. superfl.

*Blenocoes longiflora* Raf., Fl. Tellur. 3: 76 (1837)

*Nicotiana militaris* L., Sp. Pl. ed. 2: 259 (1762), pro syn. [6]

## Key to *Nicotiana* in the flora of Egypt

1. A shrub or small tree up to 6 meters, with yellow flowers........................................ *N. glauca*
2. Herb
  A Basal leaves sessile, lanceolate collapsing........................................................*N. plumbaginifolia*.
  B Basal leaves petiolate, cordate to ovate x.  Corolla lobes yellow-green ...........*N. rustica                                                                                                                                                                                xx. Corolla lobes reddish....................N. glutinosa*

## Description

*Nicotiana glutinosa* is a course, viscid-pubescent annual herb, or short-lived perennial shrub up to 2.5 m high. Stem thick, erect; branches stiff and narrowly divergent. Blades of the basal leaves are cordate-triangular to broadly cordate, 5–20 × 2-13 cm with basal lobes turned upward, an acute apex, often twisted, and the petioles shorter than the blades. Upper leaves are less markedly cordate, with rounded or cuneate bases, leaf blades 3–12 ×2–10 cm wide; petiole 1–10 cm long. Flowers are arranged in a simple racemose inflorescence; pedicels robust, 0.5–1.2 cm long. Calyx campanulate, connate, tube 0.5–0.7 cm long, lobes markedly unequal, 1–3 times as long as the tube, triangular at the base, linear to lance-linear above, usually recurved but can be erect and attenuated. Corolla 3–3.5 cm long, tube 2–2.5 cm long(with the lower end narrow and the upper end wide), white, greenish-yellow, or reddish at the throat. Limb 0.8–1.2 cm diameter, densely viscid-pubescent, with lobes broadly ovate, the tips short, acuminate, and usually recurved and channeled. Stamens slightly exserted from the throat but shorter than the limb; filaments sparsely hirsute on the lower half and oriented against the adaxial side of the corolla tube near the tips. Fruit capsule, broadly ovoid, 1.0–1.6 cm long (including calyx lobes). Seeds angular to broadly ovate, ca. 0.6 mm long, dark brown, e reticulate-foveolate with wavy ridges.

## Distribution

*Nicotiana glutinosa* is native to Bolivia, the Galápagos, and Peru. It grows primarily in the seasonally dry tropical biome [6]. *N. glutinosa* ranges from the coast of the northern half of Peru along the western flank of the outer Cordillera into southern Peru, characteristic of warm, arid areas [7]. The global taxonomic resource for Solanaceae [8] recorded 28 preserved specimens from Bolivia, Peru, and Ecuador (some are reserved in the United Kingdom and the United States of America). The Southwestern Environmental Information Network (SEINet) documented 12 records from Latin America and four in the United States of America (Table 1) that inhabit gardens and greenhouses [9]. On the other hand, the Global Biodiversity Information Facility documented 380 records (Appendix 1): 190 specimens recorded in Peru, 35 in Ecuador, 28 in Afghanistan, 19 in Bolivia, 14 in the United States of America, 6 in Germany, 5 in the Netherlands, 4 in Spain, 2 in Brazil, 2 in New Zealand, 2 in the United Kingdom, only one in France, Italy, and Portugal, and 70 records with unknown locations [10]. Some of the specimens examined that Darwin, Edmonstone, and Andersson collected from the Galapagos Islands [11].

*N. glutinosa* has not been recorded as part of the floral composition of Egypt nor neighboring countries in any previous study (Täckholm [12, 13]; Boulos[14]; El-Hadidi & Hosni [15] in Egypt; Chaudhary [16] in Saudi Arabia; Jafri El-Gadi [17] in Libya; Zohary [18] in Palestine).

**Table 1:** *Nicotiana glutinosa* herbarium sheets according to the SEINet Portal Network [9]

Symbiota ID	Collector	Date	Country	Locality	Habitat
23,683,671	C. Schweinfurth	1915	USA	Bussey Institution Forest Hills	Garden
23,683,673					
23,683,675	T. C. Plowman	1976		-	
7,881,545	Goodspeed	1963		N.C. State College	Greenhouse
28,292,664	Charles M. Rick; M. O. Rick	1970	Ecuador	Ecuador: Province Loja. Slopes of LaToma on the highway to Loja	*-*
15,528,795	Barbara Pickersgill	1971	Peru	Ayacucho Basin. 11 km. from Ayacucho on road to Huancayo	Roadside
21,398,917	-	-		-	-
8,112,089	Farruggia, F.T	2010		Between Nansha (Rupe) and Contumaza, ~ 2 km from Rupe	Bridge at creek roadside, dry spiny shrublands
28,302,407	Charles M. Rick	1956		Peru: Department Cajamarca. 18 km E of Chongoyape. Tributary of Río Chancay	-
28,302,417	Charles M. Rick	1956		Peru: Department Cajamarca. Along the road between Tambo and Cascas. Almost 2 km	The population of several plants, about 1.5 m
28,302,525	Charles M. Rick	1957		Peru: Department Lima. 6 km northeast of Churin at a location of landslide blocking	Scattered along the roadside for 15 km below Churin. Plants very abundant
19,409,576	A. Sagástegui Alva, M. O. Dillon, et al	1984		Abajo de Raquia (Ruta a Pativilca-Huaraz)	Borde de carretera

## Materials and methods

### Study area

**Fig. 1:**
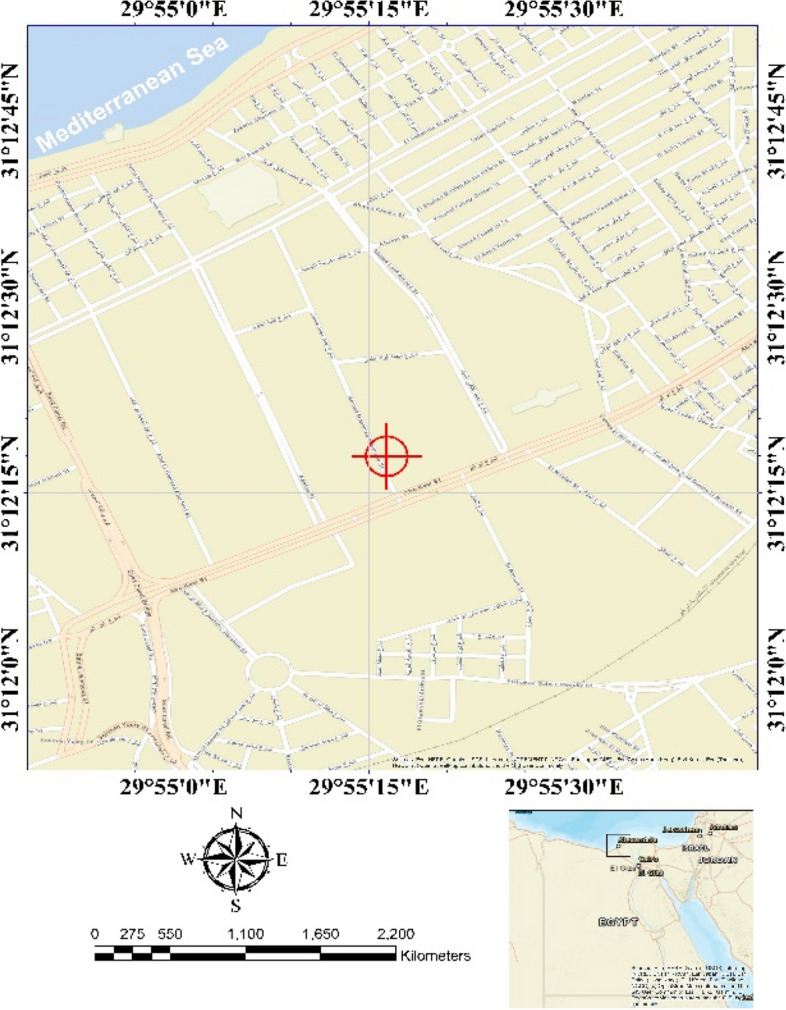
Study area location in Alexandria (  )

The current study is conducted in Alexandria, Egypt, on the Mediterranean Sea coast (Figure 1). The city of Alexandria is located to the west of the Rosetta branch of the Nile River. Alexandria city has a unique climate compared to the surrounding areas and the inland deserts of Egypt, with a temperate summer and high humidity, especially in the warmest months, July, and August. Winter is cool with regularly strong storms, usually accompanied by heavy rain. The average maximum air temperature of 36.6 ^◦^C is recorded during summer, and a minimum average of 8.2 ^◦^C during winter. The mean annual amount of precipitation is 178.63 mm, with a rate of 5 mm per rainy day. The average annual wind speed ranges from −3.03 kt to 1.28 kt, increasing at a rate of 0.125 kt/year [19].

The famous Mediterranean port city of Alexandria was established in B.C. 332 by Alexander the Great [20]. Alexandria City has rich historical, and cultural layers extending below sea level, and it is considered the most popular summer resort for Egyptians [21]. Besides, many historic buildings and monuments may be dated back to 331 B.C. [22]. The study area is a newly discovered archaeological site below a demolished university building.

### Field survey and data collection

From April 2020 to June 2022, ten sampling sites representing the studied species were selected in the study area. The study area is located at latitude 31.205 N and longitude 29.921 E with an elevation of 6 m above sea level Figure 1.

The fieldwork was conducted at intervals to ensure the collection of plant material at different phenological stages, especially at the flowering and fruiting stages. Plant material was collected following the relevant national regulations and the international guidelines of the IUCN [23] and the Convention on the Trade in Endangered Species of Wild Fauna and Flora (CITES) [24]. Permission to collect the plant specimens of the species under investigation for scientific purposes was obtained from the Department of Botany and Microbiology at the Faculty of Science, Alexandria University. A voucher specimen was deposited in the ALEX Herbarium at Alexandria University (herbarium acronyms follow Thiers [25]) at the Faculty of Science and the herbarium collection of Alexandria University Botanic Garden (Heneidy et al. collection, deposition number 5502-5504; Figure 9.

The authors conducted identification of the species and checked by Professor Dr. Adel El-Gazzar, Professor of Plant Taxonomy at Arish University in Egypt, according to the available sources and flora [6, 11, 12, 14, 15, 17, 18, 26]. Several visits were made to Egyptian herbaria to ensure the existence of *N. glutinosa* (e.g., Alexandria University (ALEX), Assiut University (ASTU), Ain Shams University (ASUE), South Valley University (ASW), Cairo University (CAI), the Desert Research Center, Mataria (CAIH), the Agricultural Research Center (CAIM), the National Research Center (CAIRC), Mazhar Botanical Garden (MAZHAR), Suez Canal University (SCUI), and Tanta University (TANE), herbarium acronyms follow Thiers [25]. No preserved herbarium specimens of *N. glutinosa* were found.

### Leaf morphology

Mature leaves were examined using a stereomicroscope (National DC3-420T Digital Microscope) and photographed using Xiaomi Mi Note 10 Lite cameras. Leaf trichomes were examined by an OPTIKA C-B 10 light microscope (LM) and photographed using an OPTIKA am B 10 digital camera. For Scanning Electron Microscope (SEM) investigation, parts of the leaf were mounted onto SEM stubs, coated with gold, and examined and photographed using a JEOL JSM-IT100 SEM. The SEM photographs were carried out at the Institute of Nanoscience and Nanotechnology, Kafrelsheikh University. The terminology was followed as given by Crang et al. [27].

### Stem anatomy

Samples were taken from the main stem at 10 cm above the ground, and a 3–4 cm segment was placed in the holder of a LEICA RM2125 RTS-Microtome for sectioning. The samples were sectioned in the transverse (cross-section) direction, with a 15–20 µm thickness. Each section was then transferred to a glass slide, stained with a Safranin-Astra blue mixture, dehydrated with ethanol (96% and dehydrated ethanol), and rinsed with xylol to prepare for mounting in Canada balsam [28]. All micro-sections were oven-dried at 60 degrees Celsius for 24–48 hours before being examined under an Olympus B41X microscope for micro-photos with a Canon EOS 1200D camera and EOS utility software. According to Crivellaro & Schweingruber [29].

### Pollen grain morphology

Flowers were fixed in 70% ethanol for light microscopy investigation. Mature anthers from the collected flowers were left to dry, carefully opened using sharp needles, and sputtered onto glass slides. The pollen grains (25) were examined by an OPTIKA C-B 10 microscope and photographed using an OPTIKA am B 10 digital camera. Polar axis (P) and equatorial diameter (E) were measured; pollen size, shape, and aperture type were also assessed. For scanning electron microscope investigation, non-acetalized pollen grains were transferred onto a metallic stub using double-sided and coated with a thin layer of gold in a sputtering chamber, then scanned and photographed using a JEOL JSM-IT200 SEM for exine and aperture ornamentations. The terminology used for describing pollen grain morphology was followed as given by [30], Punt et al. [31], and Hesse et al. [32].

### Seed morphology

Mature seeds (20 seeds) were examined using a stereomicroscope (National DC3-420T Digital Microscope) and photographed using Xiaomi Mi Note 10 Lite cameras. Seed color, length, and width, as well as hilum position, were studied. For SEM investigation, the mature seeds were mounted onto SEM stubs, coated with gold, and examined and photographed using a JEOL JSM-IT200 SEM. The terminology was followed as given by Barthlott [33] and Stearn [34]. SEM photographs were carried out in the Electron Microscope Unit, Faculty of Science, Alexandria University.

### Size structure

The population structure of *N. glutinosa* was evaluated in terms of size distribution. For achieving this, the height (H) and mean crown diameter (D) of each of the 1029 individuals in the ten stands were measured, based on 2-4 diameter measurements per individual, and its volume was calculated as a cylinder according to the following equation: Volume= π r^2^ H; where r is the radius and H is the individual's height. The size index of each individual was calculated as the average of its height and diameter {(H+D)/2}. The size index estimates were then used to classify the population into ten size classes: (I) ≤10, (II) >10-20, (III) >20-30, (IV) >30-40, (V) >40-50, (VI) >50-60, (VII) >60-70, (VIII) >70-80, (IX) >80-90, (X) >90 The first 3 classes represent the juveniles [35]. Also, the mean height, diameter, size index, v6olume, and height to diameter ratio with size class were assessed [36]. The number of *N. glutinosa* individuals in each stand was counted and then used to calculate density.

### Herbarium sheets and drawings

Samples of *N. glutinosa* were selected for hand drawing. Herbarium specimens of the studied species were collected, identified, and deposited at the Botanic Garden Herbarium (BGH) (Heneidy et al. Collection) of the Faculty of Science, Alexandria University

## Results

### Leaf morphology

**Fig. 2:**
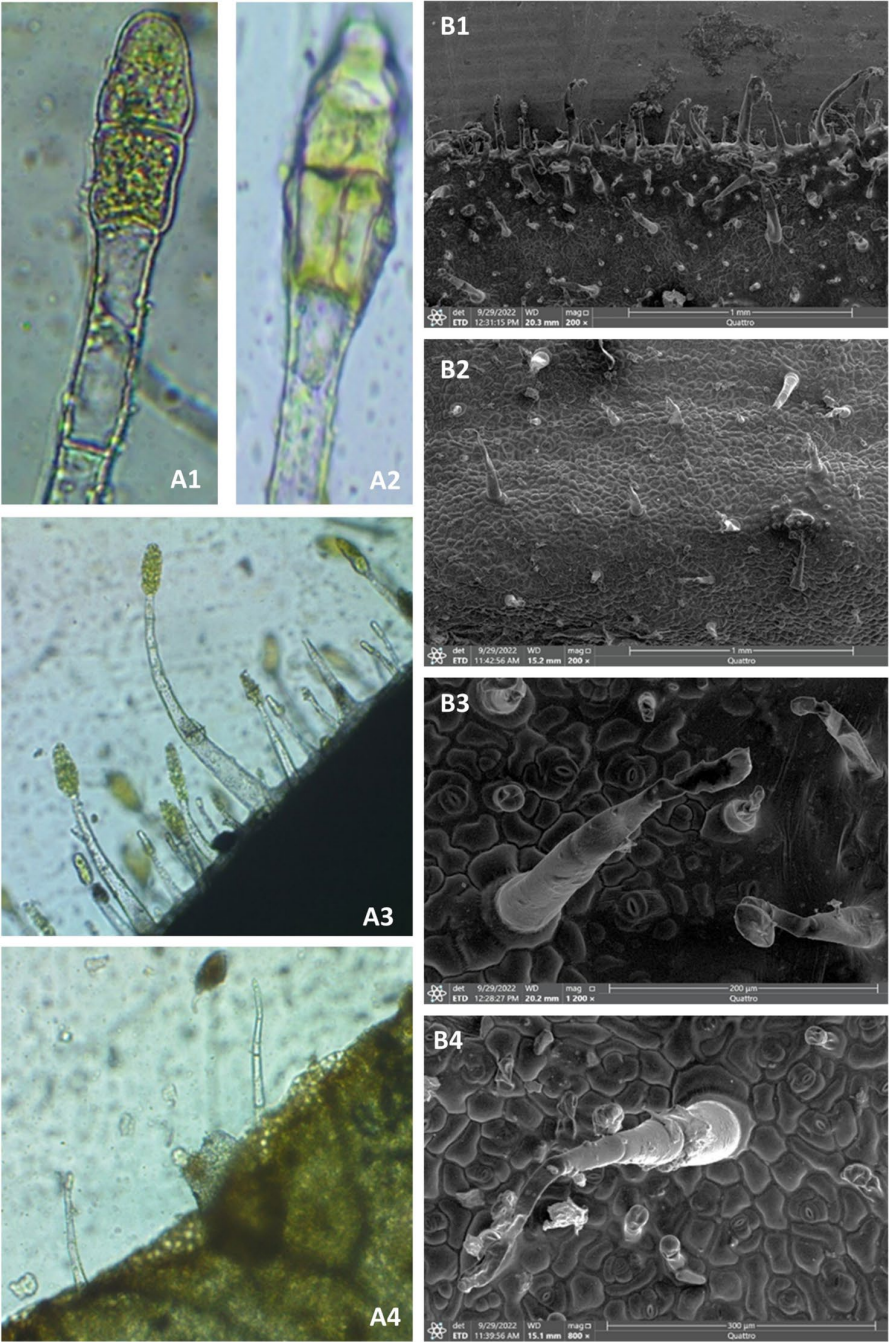
Micrographs of leaf surfaces of *Nicotiana glutinosa* under light microscope (**A**): A1 glandular trichome with two secretory cell heads, A2 with four cell heads, A3 lower epidermis, and A4 upper epidermis. SEM micrographs (**B**), upper epidermis B1 and B3, and lower epidermis B2 and B4

Mature leaves of *Nicotiana glutinosa* were examined under a stereomicroscope, compound light (LM), and scanning electron microscope (SEM) for both upper (adaxial) and lower (abaxial) surfaces Figure 2. The leaves have anomocytic stomata that lack subsidiary cells with raised guard cells which occur above the level of the adjacent epidermal cells. The upper and lower epidermis are covered with multicellular uniseriate glandular hairs either with long or dwarf stalks, but the lower surface is densely covered with multicellular uniseriate glandular trichomes. In contrast, the lower surface is sparsely covered with multicellular uniseriate non-glandular trichomes. The head of glandular trichomes may have two or four secretory cells.

### Stem anatomy

**Fig. 3:**
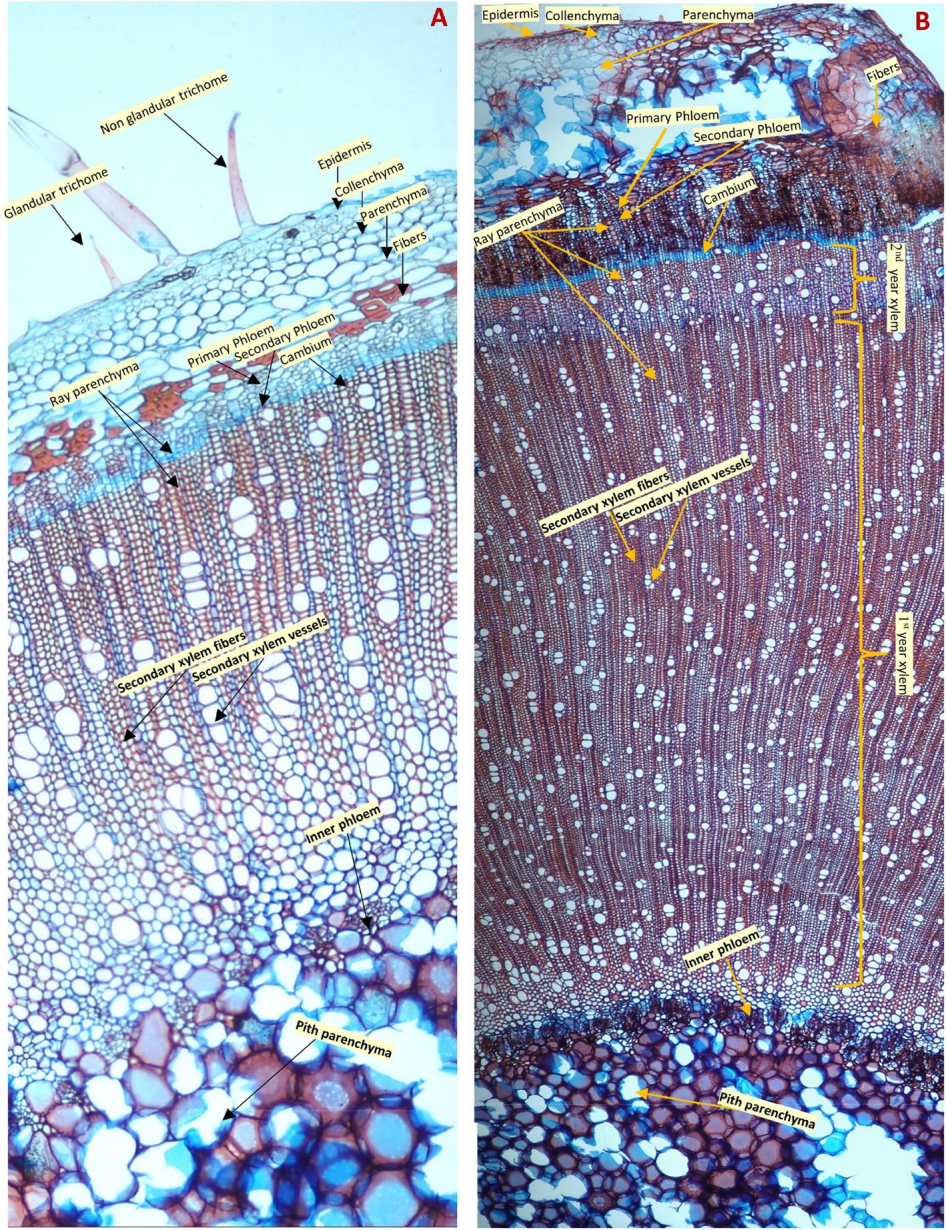
Stem anatomy of *Nicotiana glutinosa* (**A**) A one-year-old; (**B**) A two-year-old

The transverse section (T.S.) of the *N. glutinosa* stem indicates a complete ring of vascular tissue with diffused porous xylem and vessels in radial multiples (2–12) Epidermis with both glandular and non-glandular multicellular uniseriate trichomes. The cortex comprises angular collenchyma, spherical parenchyma, and some patches of fibers supporting the primary phloem. The pith is composed mainly of parenchyma with a little patch of fiber supporting the intraxylary (inner) phloem. Some parenchyma contains crystal sand. A transverse section of a one- and two-year-old plant is presented in Figure 3.

Pollen grain morphology

### Pollen grain morphology

**Fig. 4:**
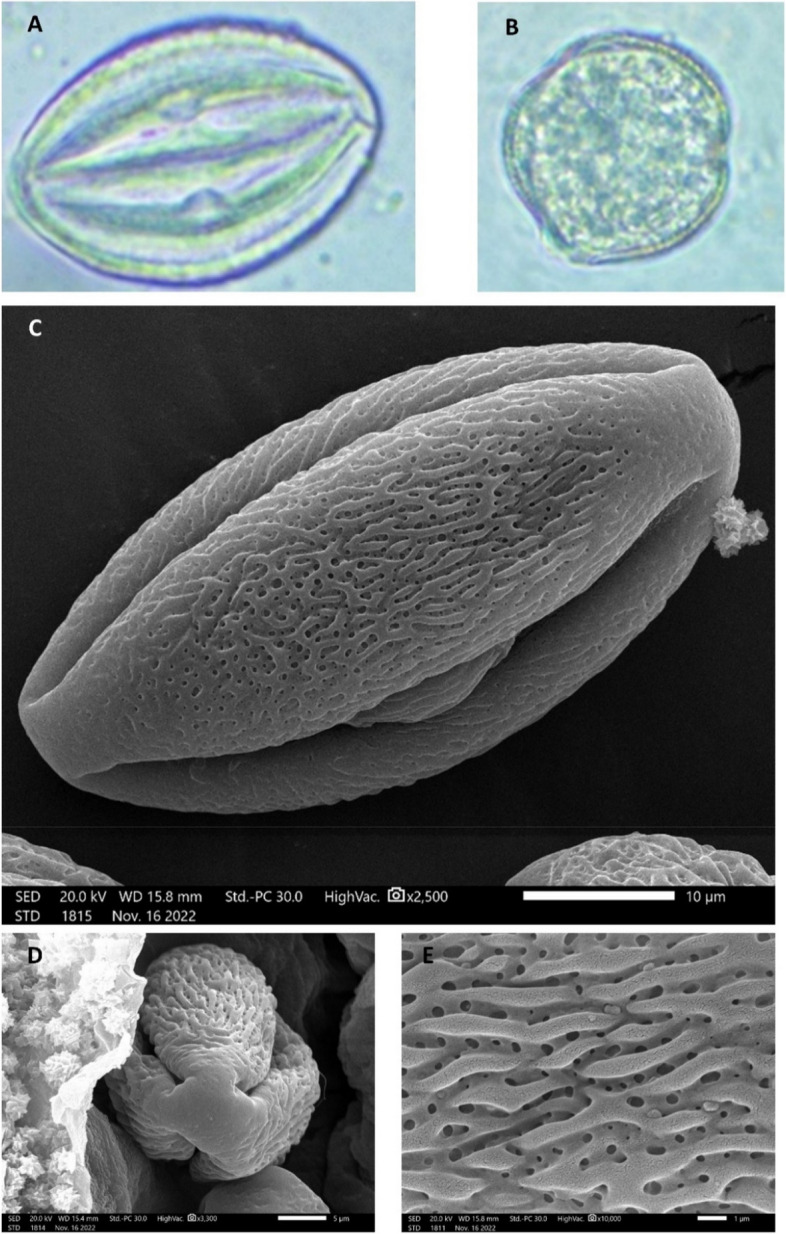
Micrographs of *Nicotiana glutinosa* pollen grains. (**A**–**B**) LM micrographs: (**A**) equatorial view, (**B**) polar view. **C**–**E** SEM micrographs: (**C**) equatorial, (**D**) polar view, and (**E**) exine ornamentation SEM

Pollen grains are monads as they do not remain united in tetrads at maturity but dissociated into a single pollen grain, isopolar and radially symmetric, perprolate in the equatorial view (i.e., the grain with a polar axis that is greater than the equatorial diameter in a ratio of approximately 2: 1), and semicircular in the polar view (i.e., The length of the vertical axis and horizontal axis are approximately equal). (Figure 4). Pollen of large size, with a P axis ±53.9 µm and an E axis ±26.2 µm. The pollen grain has a large apocolpium, tri-angul-colporate apertures, long fusiform colpi with acute ends, an ornamented membrane, and tenuimarginate. Exine sculpture Rugulate- perforate as elongated sculpturing elements are greater than 1μm long, pattern irregularly arranged, and surface having small holes or depressions less than 1μm in diameter.

### Seed morphology

**Fig. 5:**
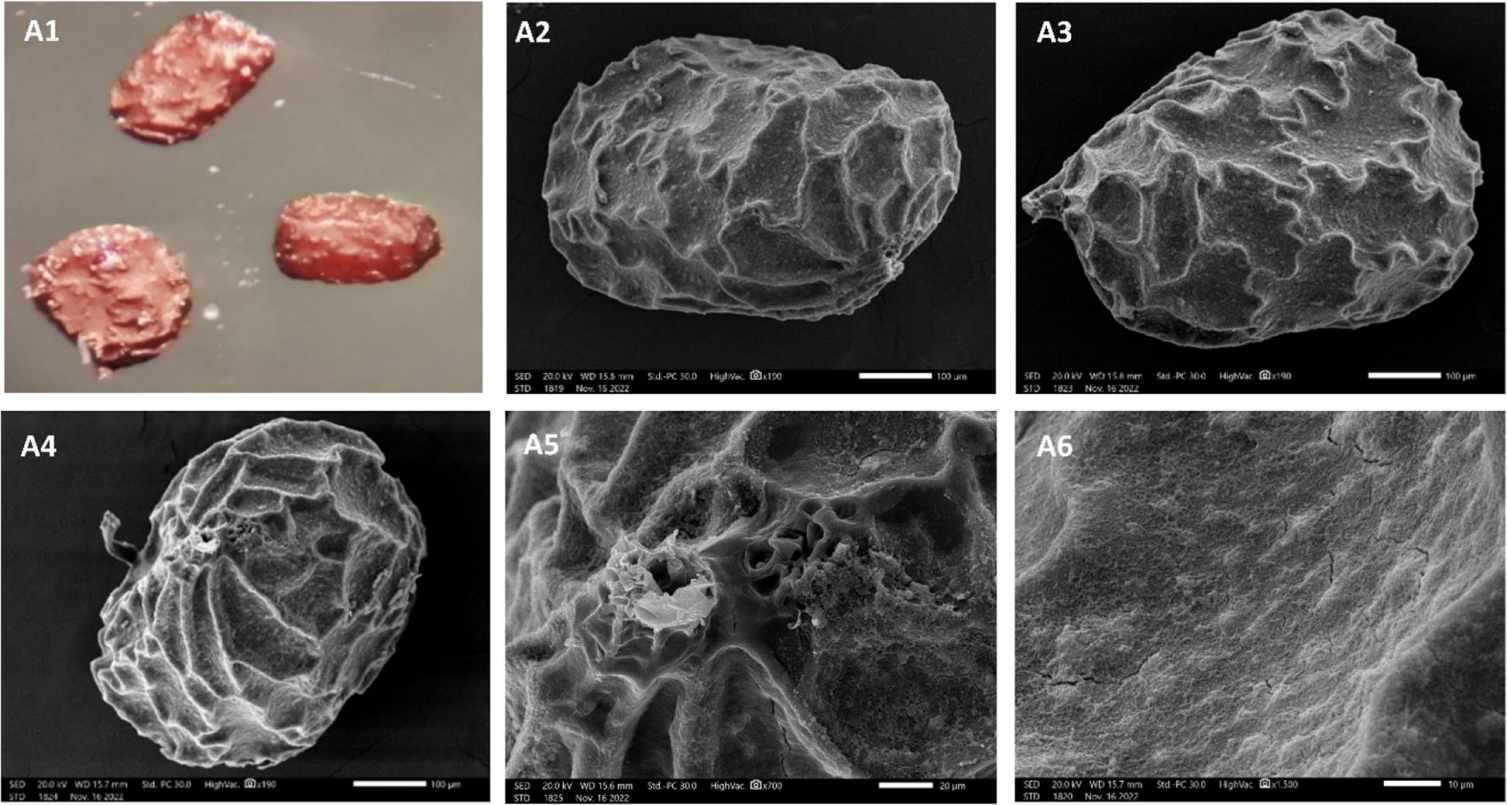
Stereomicrographs of *Nicotiana glutinosa* seeds (A1) taken with a stereomicroscope (A2-A6 SEM). A4 hilum position, A6 seed epidermis

Mature seeds (20 seeds) of *N. glutinosa* were examined and photographed (Figure 5). The seeds were brown in color and range from ovate to oblong-ovate (210 × 146 µm) with a basal raised hilum. The surface sculpture was f reticulate. The epidermal cells were irregular in shape with an undulating, narrow, raised anticlinal wall. The periclinal walls were depressed with globulate surface sculpture.

### Plant size structure

**Fig. 6:**
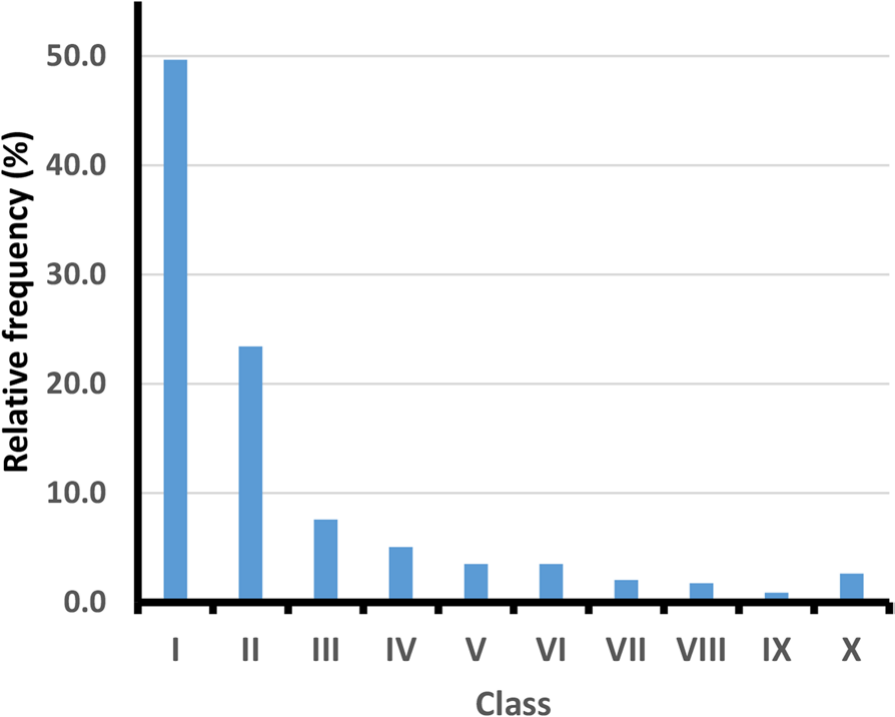
Frequency distribution of *Nicotiana glutinosa* individuals in the study area. The size classes: 1: ≤ 10, 2: > 10–20, 3: > 20–30, 4: > 30–40, 5: > 40–50, 6: > 50–60, 7: > 60–70, 8: > 70–80, 9: > 80–90, and 10: > 90 cm

**Fig. 7:**
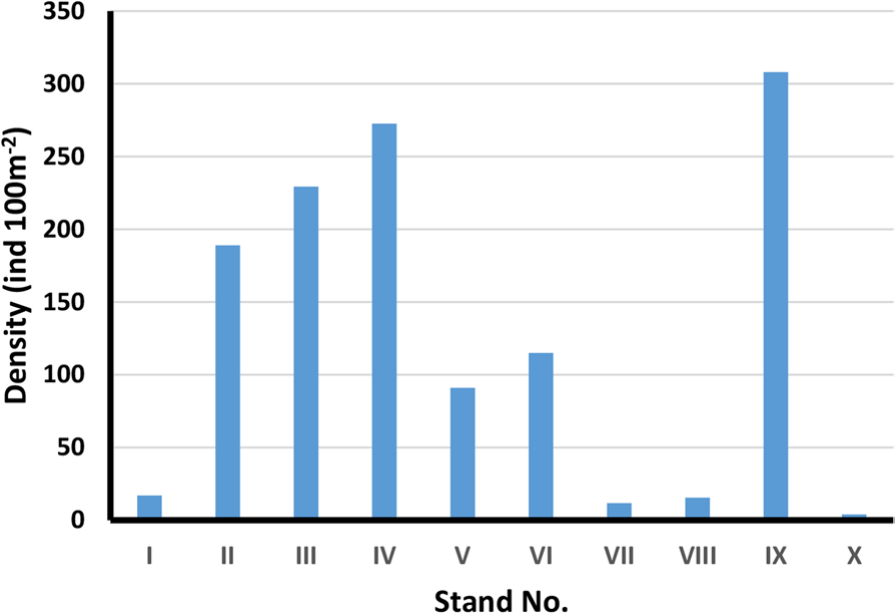
Density (ind. 100 m-2) of *Nicotiana glutinosa* in the study area

The height, diameter, size index, volume, and height/diameter ratio of *N. glutinosa* differed significantly in relation to size classes (*P*<0.001, Table 2). The number of individuals increases in the first three classes, where they represent 80% of the total sampled individuals (Figure 6). Generally, the mean crown diameter ranges from 1.33-150.00 cm, while the average height ranges from 1.00 to 160.00 cm. The mean size index ranges from 1.33 to 150.00 cm, while the average volume ranges from 0.002 to 2648.38 dm^3^. On the other hand, the density ranges from 4–273 individuals /100 m^-2^ Figure 7.

**Table 2:** Minimum – Maximum of the dimensions in relation to size classes of *Nicotiana glutinosa.* The size classes: (I) ≤ 10, (II) > 10–20, (III) > 20–30, (IV) > 30–40, (V) > 40–50, (VI) > 50–60, (VII) > 60–70, (VIII) > 70–80, (IX) > 80–90, (X) > 90 cm. analysis of data done by using SPSS [37]

class	No. of Ind	D (cm)	H (cm)	Index (cm)	Volume (dm^3^)
I	511	1.33–11.67	1.00–15.00	1.33–10.00	0.002–0.85
I I	241	5.00–26.67	8.00–26.00	10.50–20.00	0.47–5.98
I I I	78	6.33–47.67	9.00–50.00	20.33–30.50	1.32–21.81
IV	52	8.33–66.67	9.00–60.00	30.67–40.00	3.27–55.64
V	36	8.67–53.67	30.00–80.00	40.17–50.00	4.54–90.44
VI	36	11.67–62.33	39.00–95.00	50.33–60.00	10.15–160.27
V I I	21	23.33–77.00	60.00–110.00	60.83–68.50	47.01–279.26
VI I I	18	46.67–95.00	50.00–110.00	70.83–80.00	170.96–393.91
IX	9	22.00–103.33	75.00–155.00	80.83–90.00	58.89–628.65
X	27	61.67–150.00	90.00–160.00	90.83–150.00	358.22–2649.38
**Total**	1029	**1.33–150.00**	**1.00–160.00**	**1.33–150.00**	**0.002–2649.38**
**F-value**		852.59***	1511.69***	5842.72***	462.19***

^***^significance at ≤ 0.001

### Species collection, herbarium sheets, and drawings

**Fig. 8:**
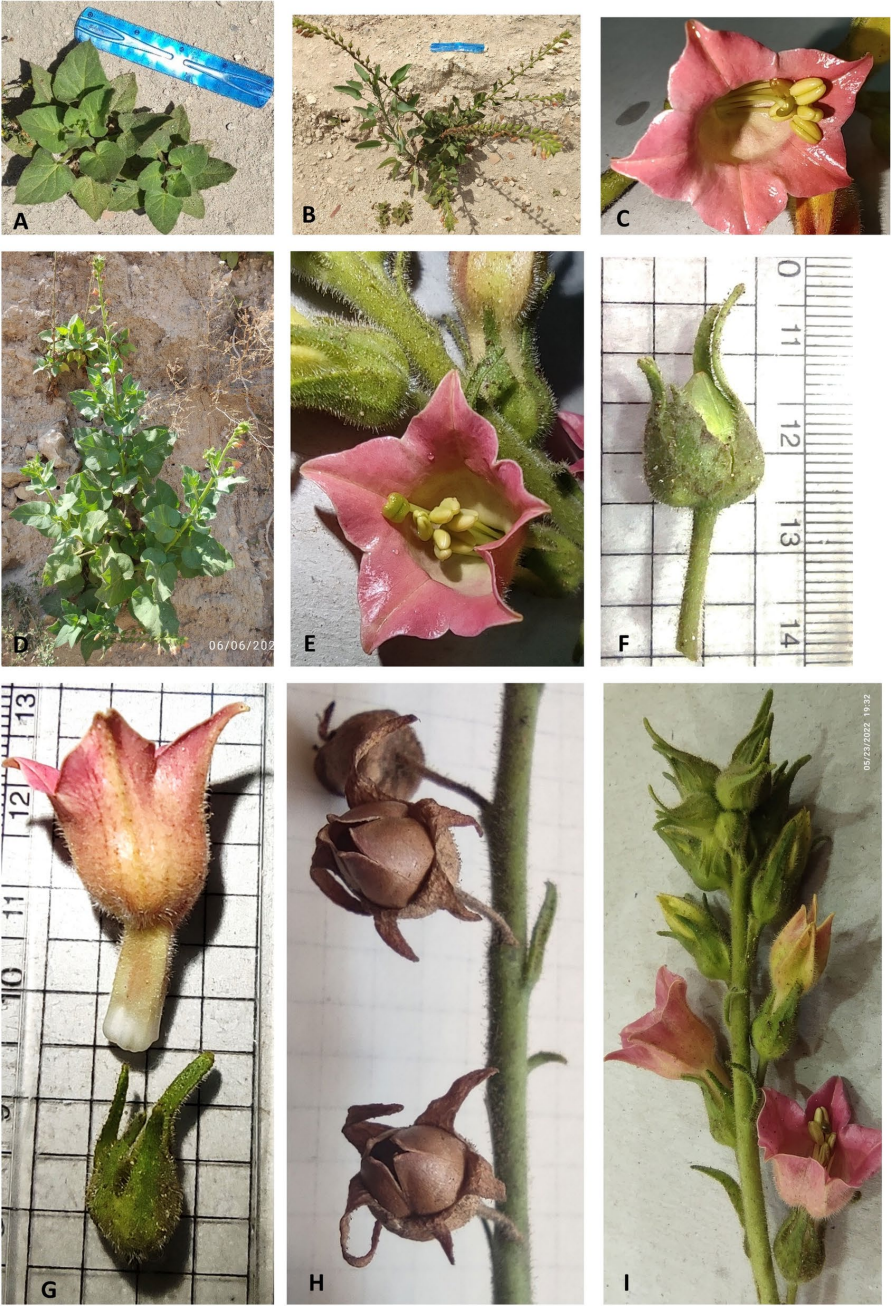
*Nicotiana glutinosa* habits and habitats (**A**) seedling, (**B**) flowering and early fruiting, (**D**) early flowering stage, (**C**) & (**E**) corolla with stamens and exerted green stigma, (**F**) persistent calyx, (**G**) detached calyx and corolla, (**H**) septifragal capsule, and (**I**) flowering branch, photo by S. M. Toto

**Fig. 9:**
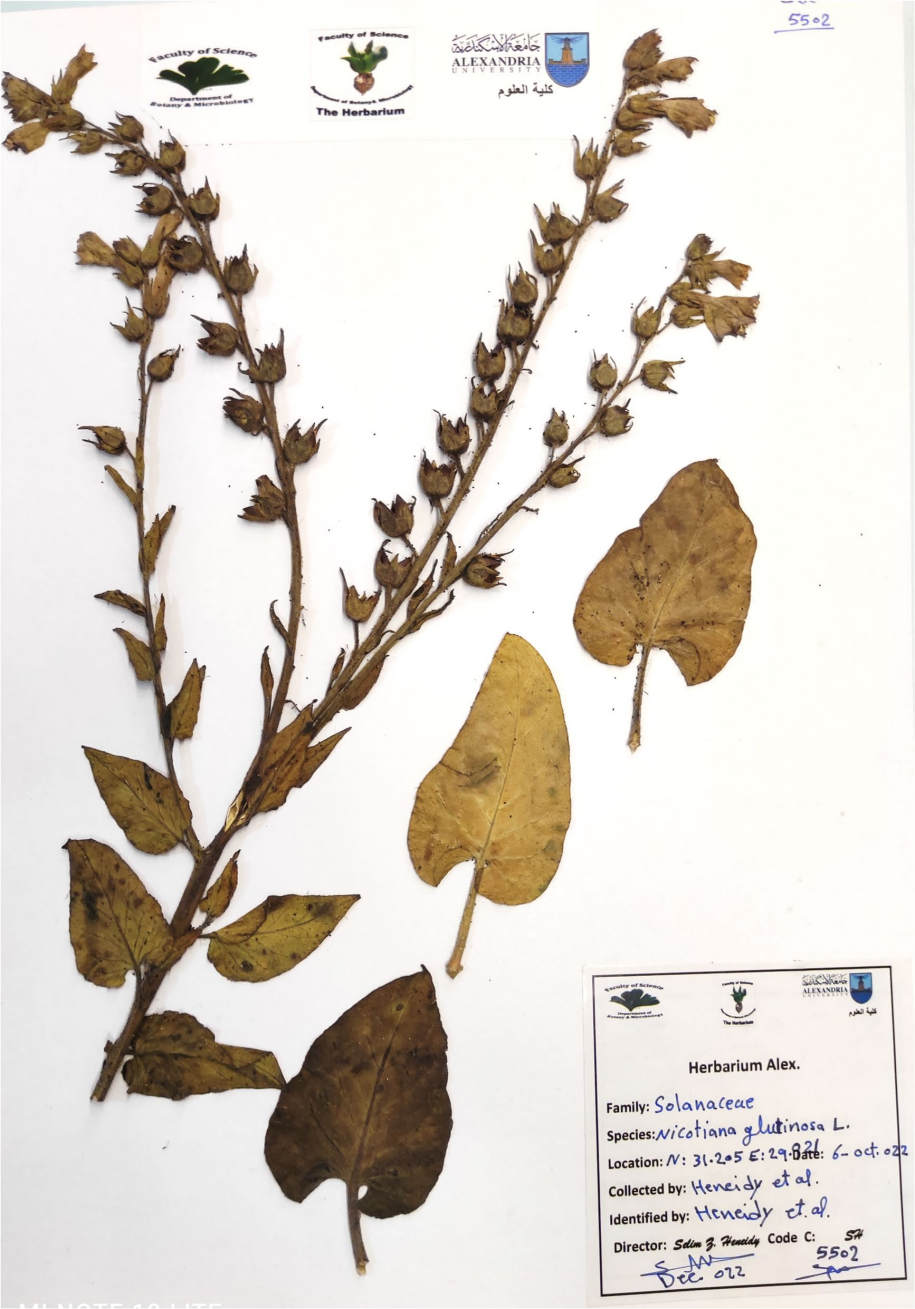
Voucher herbarium specimen of *Nicotiana glutinosa* deposited in (BGH)

**Fig. 10:**
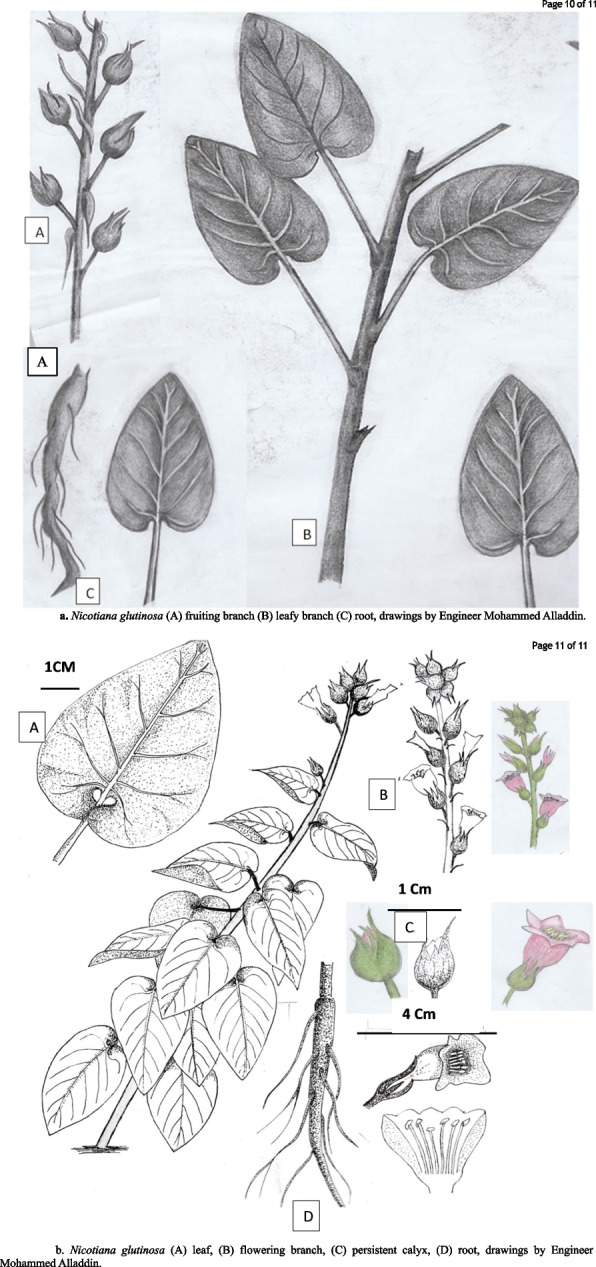
**a. ***Nicotiana glutinosa* (**A**) fruiting branch (**B**) leafy branch (**C**) root, drawings by Engineer Mohammed Alladdin. **b**. *Nicotiana glutinosa* (**A**) leaf, (**B**) flowering branch, (**C**) persistent calyx, (**D**) root, drawings by Engineer Mohammed Alladdin

The study species were monitored throughout the year; the plants produced flowers. The different plant stages and parts photographed using the Nikon Coolpix AW100 camera are demonstrated in Figure 8. Voucher specimens were deposited in the Herbarium of Alexandria University (ALEX) Faculty of Science, and another specimen was processed to make herbarium specimens at the Herbarium of the Botanic Garden (Heneidy et al. Collection, deposition number 5502) (Figure 9). Hand drawing of the study species with scale for the whole plant and its parts (Figures 10 a and b).

## Discussion

This study adds *Nicotiana glutinosa* as a new record belonging to the Solanaceae, to the flora of Egypt. The species was found in a newly discovered archaeological excavation site just below a demolished university building in Alexandria. The urban flora in Alexandria city has been thoroughly surveyed by Heneidy et al. [21, 38], but *N. glutinosa* is detected only in this sole site. The Egyptian flora is well documented in many references, such as Täckholm [12, 13] and Boulos [14, 26]. It comprises 2145 species and 220 infra-specific taxa of native and naturalized vascular plants, in addition to 175 species and subspecies of mosses and 13 species of hepatics [39, 40]. A precise revision of the flora of Egypt and the flora of neighboring countries (flora of Saudi Arabia, flora of Palestine, flora of Libya) and online analytical florae of neighboring areas revealed that *N. glutinosa* is not recorded in Egypt nor any of the nearby countries.

From the floristic point of view, if any species has not been recorded in the flora of an area for the last 50 years, it will be considered a new record species [41]. According to this concept, *N. glutinosa* is considered a newly recorded species in Egypt, as it has not been recorded in the flora of Egypt for the last 50 years [12–15, 26, 42]. The authors did not recognize any records and observations of *N. glutinosa* in any study on the Egyptian flora during this period. This is also supported by several visits to the herbaria in Egyptian institutes (11 of the largest Egyptian herbaria; Alexandria University (ALEX), Assiut University (ASTU), Ain Shams University (ASUE), South Valley University (ASW), Cairo University (CAI), the Desert Research Center, Mataria (CAIH), the Agricultural Research Center (CAIM), the National Research Center (CAIRC), Mazhar Botanical Garden (MAZHAR), Suez Canal University (SCUI), and Tanta University (TANE), herbarium acronyms follow Thiers [25]) to ensure the existence of *N. glutinosa*, which indicated that no preserved herbarium specimens of the study species are vouchered in these places. Only five species of *Nicotiana* (*N. glauca* R. C. Graham*, N. rustica* L*., N. plumbaginifolia* Viv*., N. alata* Link & Otto*, and N. tabacum* L*.*) were found as voucher herbarium specimens deposited in herbaria of Egypt. However, none possess the newly recorded species (*N. glutinosa*). The distribution of *N. glutinosa* is restricted to Latin America, this is supported by a survey conducted by the global taxonomic resource for Solanaceae [8], which recorded 28 preserved specimens (some of which are reserved in the United Kingdom and the United States of America), all collected from Bolivia, Peru, and Ecuador, as well as the Southwestern Environmental Information Network (SEINet), which documented 12 records all from Latin America, including four records in the United States of America but not in natural habitats, while the Global Biodiversity Information Facility documented 380 records in Europe, Asia, and America but still no single record in Egypt and its neighbors. The plant population structure is usually the result of the effects of biotic and abiotic factors on individuals' growth and mortality rates [43]. Results of the present study revealed that *N. glutinosa* has successfully established itself in one of the archaeological sites in Egypt, showing a “healthy” population with a high degree of size inequality, which could indicate self-replacing populations. Generally, our study's population of *N. glutinosa* is characterized by the relative majority of juvenile (small-sized) individuals, particularly in depressed and flat areas. On the contrary, slopes supported a much smaller number of juvenile individuals. Feeley et al. [44] reported that a species population with a very high density of juveniles would not necessarily produce more reproductive adults than a population with a few small-sized individuals. The size structure of a plant population has been frequently used to assess regeneration status and predict future population changes [45], which is a good measure for the future trend of establishing this species in the study area as a regional habitat. From our observations during the field study, *N. glutinosa* seems to have invasive potential, as it shows characteristics shared by most invasive species that are thought to help in their successful establishment in new habitats. For example, it shows fast growth and maturity, abundant seed production, and highly successful seed dispersal, germination, and colonization. The examined mature stems of *N. glutinosa* were composed of one or two successive rings of xylem, indicating one- and two-year-old plants. We presume the introduction of *N. glutinosa* to Egypt may have been very old, as it was recorded by our research team only in a newly discovered archaeological site just below a demolished university building in Alexandria City. We did not find any evidence regarding a time frame or way of dispersal for this species. From our field observations, this species was not recorded at other nearby sites. We presume that seeds were buried in the soil a long time ago, and when the soil was re-excavated and worked in these places and conditions were suitable, the plant reappeared strongly and began rapidly spreading. It is worth mentioning that the presence of tobacco in Egyptian mummies and the evidence for the use of nicotine-derived insecticides at least since the late 18th century may [46] provide a much more probable explanation. It is hoped that the current study will highlight the importance of monitoring introduced species for early detection of invasion potential and minimizing risks to native plant diversity. Besides, the economic and medicinal importance of the studied plant (*N. glutinosa*) highlights the need for further studies on the distribution, sociability, and invasiveness of the plant. Moreover, careful records of its presence, distribution, and abundance in the different habitats of Egypt and neighboring countries will be recommended.

## Conclusions and Recommendations

The study revealed that *Nicotiana glutinosa* has successfully established itself in the archaeological sites of Egypt. The species should be subjected to continuous monitoring to assess its behavior in the wild habitat in Egypt. Further surveys should be pursued elsewhere in Egypt to assess the species occurrence and rate of expansion. Detection of the spatial distribution and evaluation of the rate of expansion of *N. glutinosa* based on continuous under-control field observation and monitoring.

### Supplementary Information

**Supplementary material 1**.

## Data Availability

“The datasets used and analyzed during the current study are available from the corresponding author on reasonable request.”
