# Supplementary material for: Biology of Nicotiana glutinosa L., a newly recorded species from an archaeological excavation site in Egypt
Source: BMC Plant Biol. 2024 Feb 28;24:148. doi: 10.1186/s12870-024-04816-z (PMC10900750; doi:10.1186/s12870-024-04816-z)
Supplement: Supplementary file 1 — Supplementary material 1. [file 12870_2024_4816_MOESM1_ESM.docx]

**Appendix 1:** The 380 records of *Nicotiana glutinosa,* all over the world according to Global Biodiversity Information Facility (GBIF) *.

| No. | GBIF ID | Country | Year of collection | State of specimen |
| --- | --- | --- | --- | --- |
| 1 | 41234586 | United States of America |  | Occurrence |
| 2 | 41234589 | United States of America |  | Occurrence |
| 3 | 41234662 | United States of America |  | Occurrence |
| 4 | 41234666 | United States of America |  | Occurrence |
| 5 | 41234668 | United States of America |  | Occurrence |
| 6 | 41234678 | United States of America |  | Occurrence |
| 7 | 768796765 | United States of America | 1970 | Preserved specimen |
| 8 | 1852137564 | United States of America | 1966 | Preserved specimen |
| 9 | 1930865397 | United States of America | 1982 | Preserved specimen |
| 10 | 1990217427 | United States of America |  | Preserved specimen |
| 11 | 1998469493 | United States of America | 1915 | Preserved specimen |
| 12 | 1998576760 | United States of America | 1915 | Preserved specimen |
| 13 | 1998685336 | United States of America | 1976 | Preserved specimen |
| 14 | 2900350083 | United States of America | 1918 | Preserved specimen |
| 15 | 1057402487 | United Kingdom | 1755 | Preserved specimen |
| 16 | 1135487888 | United Kingdom | 2004 | Preserved specimen |
| 17 | 1142654615 | Spain |  | Human observation |
| 18 | 1142654670 | Spain |  | Human observation |
| 19 | 1825736122 | Spain | 1833 | Preserved specimen |
| 20 | 1936082364 | Spain |  | Preserved specimen |
| 21 | 2598865444 | Portugal | 1966 | Preserved specimen |
| 22 | 317852539 | Peru |  | Preserved specimen |
| 23 | 317852540 | Peru |  | Preserved specimen |
| 24 | 317852541 | Peru |  | Preserved specimen |
| 25 | 438578952 | Peru |  | Preserved specimen |
| 26 | 438912465 | Peru |  | Preserved specimen |
| 27 | 438912466 | Peru |  | Preserved specimen |
| 28 | 438912467 | Peru |  | Preserved specimen |
| 29 | 474629031 | Peru |  | Preserved specimen |
| 30 | 474715572 | Peru |  | Preserved specimen |
| 31 | 575013138 | Peru | 2002 | Preserved specimen |
| 32 | 575123578 | Peru | 2002 | Preserved specimen |
| 33 | 575236335 | Peru | 2006 | Preserved specimen |
| 34 | 872338251 | Peru |  | Preserved specimen |
| 35 | 991746338 | Peru | 2013 | Preserved specimen |
| 36 | 991746410 | Peru | 2013 | Preserved specimen |
| 37 | 991746687 | Peru | 2013 | Preserved specimen |
| 38 | 991747131 | Peru | 2013 | Preserved specimen |
| 39 | 991747501 | Peru | 2013 | Preserved specimen |
| 40 | 991747597 | Peru | 2013 | Preserved specimen |
| 41 | 991748107 | Peru | 2013 | Preserved specimen |
| 42 | 991748243 | Peru | 2013 | Preserved specimen |
| 43 | 991748917 | Peru | 2013 | Preserved specimen |
| 44 | 1050621730 | Peru | 1998 | Preserved specimen |
| 45 | 1055300790 | Peru | 2013 | Preserved specimen |
| 46 | 1055336966 | Peru | 2014 | Preserved specimen |
| 47 | 1055346914 | Peru | 1986 | Preserved specimen |
| 48 | 1055549189 | Peru | 2013 | Preserved specimen |
| 49 | 1055549775 | Peru | 2002 | Preserved specimen |
| 50 | 1055703888 | Peru | 2013 | Preserved specimen |
| 51 | 1055887985 | Peru | 2014 | Preserved specimen |
| 52 | 1055927052 | Peru | 2014 | Preserved specimen |
| 53 | 1056130502 | Peru | 2014 | Preserved specimen |
| 54 | 1056133941 | Peru | 2012 | Preserved specimen |
| 55 | 1056192770 | Peru | 2013 | Preserved specimen |
| 56 | 1056400234 | Peru | 1999 | Preserved specimen |
| 57 | 1056648484 | Peru | 2002 | Preserved specimen |
| 58 | 1056755238 | Peru | 2014 | Preserved specimen |
| 59 | 1056812860 | Peru | 2002 | Preserved specimen |
| 60 | 1057187248 | Peru | 2013 | Preserved specimen |
| 61 | 1057205856 | Peru | 2013 | Preserved specimen |
| 62 | 1057453626 | Peru | 2012 | Preserved specimen |
| 63 | 1057504400 | Peru | 2002 | Preserved specimen |
| 64 | 1096692327 | Peru | 1940 | Preserved specimen |
| 65 | 1096692329 | Peru | 1940 | Preserved specimen |
| 66 | 1096692332 | Peru |  | Preserved specimen |
| 67 | 1096697590 | Peru |  | Preserved specimen |
| 68 | 1135482940 | Peru | 1953 | Preserved specimen |
| 69 | 1135482959 | Peru | 1869 | Preserved specimen |
| 70 | 1144984859 | Peru | 2013 | Living specimen |
| 71 | 1144984986 | Peru | 2013 | Living specimen |
| 72 | 1144984992 | Peru | 2013 | Living specimen |
| 73 | 1212568534 | Peru |  | Preserved specimen |
| 74 | 1228171206 | Peru |  | Preserved specimen |
| 75 | 1228178813 | Peru |  | Preserved specimen |
| 76 | 1228184557 | Peru |  | Preserved specimen |
| 77 | 1228191139 | Peru | 1983 | Preserved specimen |
| 78 | 1228213216 | Peru | 1983 | Preserved specimen |
| 79 | 1228232826 | Peru | 1953 | Preserved specimen |
| 80 | 1228236983 | Peru | 1984 | Preserved specimen |
| 81 | 1228256706 | Peru |  | Preserved specimen |
| 82 | 1228262797 | Peru | 1987 | Preserved specimen |
| 83 | 1228270639 | Peru | 1993 | Preserved specimen |
| 84 | 1228274962 | Peru |  | Preserved specimen |
| 85 | 1228284549 | Peru |  | Preserved specimen |
| 86 | 1228287855 | Peru |  | Preserved specimen |
| 87 | 1228299455 | Peru |  | Preserved specimen |
| 88 | 1228303159 | Peru |  | Preserved specimen |
| 89 | 1228309283 | Peru |  | Preserved specimen |
| 90 | 1228316034 | Peru |  | Preserved specimen |
| 91 | 1228319020 | Peru | 1953 | Preserved specimen |
| 92 | 1228362153 | Peru | 1977 | Preserved specimen |
| 93 | 1228369269 | Peru |  | Preserved specimen |
| 94 | 1228370333 | Peru |  | Preserved specimen |
| 95 | 1228384813 | Peru |  | Preserved specimen |
| 96 | 1228393076 | Peru |  | Preserved specimen |
| 97 | 1228422667 | Peru |  | Preserved specimen |
| 98 | 1228432586 | Peru |  | Preserved specimen |
| 99 | 1228458174 | Peru |  | Preserved specimen |
| 100 | 1228480109 | Peru |  | Preserved specimen |
| 101 | 1228494994 | Peru |  | Preserved specimen |
| 102 | 1228499216 | Peru |  | Preserved specimen |
| 103 | 1228505915 | Peru | 1993 | Preserved specimen |
| 104 | 1228523864 | Peru |  | Preserved specimen |
| 105 | 1228525228 | Peru | 1957 | Preserved specimen |
| 106 | 1228565750 | Peru |  | Preserved specimen |
| 107 | 1228582637 | Peru |  | Preserved specimen |
| 108 | 1228603843 | Peru | 1970 | Preserved specimen |
| 109 | 1228605158 | Peru |  | Preserved specimen |
| 110 | 1228619562 | Peru | 1928 | Preserved specimen |
| 111 | 1228625229 | Peru | 1984 | Preserved specimen |
| 112 | 1228627564 | Peru | 1994 | Preserved specimen |
| 113 | 1257688273 | Peru | 2007 | Preserved specimen |
| 114 | 1257734689 | Peru | 2011 | Preserved specimen |
| 115 | 1258007215 | Peru | 2013 | Preserved specimen |
| 116 | 1258130369 | Peru | 2010 | Preserved specimen |
| 117 | 1258362822 | Peru | 1938 | Preserved specimen |
| 118 | 1258363069 | Peru | 1961 | Preserved specimen |
| 119 | 1258448289 | Peru | 1998 | Preserved specimen |
| 120 | 1258605719 | Peru | 1978 | Preserved specimen |
| 121 | 1259256262 | Peru | 1957 | Preserved specimen |
| 122 | 1259393984 | Peru | 1964 | Preserved specimen |
| 123 | 1259568004 | Peru | 1999 | Preserved specimen |
| 124 | 1259685020 | Peru | 1964 | Preserved specimen |
| 125 | 1260313744 | Peru | 1938 | Preserved specimen |
| 126 | 1260313975 | Peru | 1942 | Preserved specimen |
| 127 | 1260315466 | Peru | 1984 | Preserved specimen |
| 128 | 1260315563 | Peru | 1983 | Preserved specimen |
| 129 | 1260315835 | Peru | 1983 | Preserved specimen |
| 130 | 1260315866 | Peru | 1934 | Preserved specimen |
| 131 | 1260317379 | Peru | 1986 | Preserved specimen |
| 132 | 1260317460 | Peru | 1993 | Preserved specimen |
| 133 | 1260317706 | Peru | 1952 | Preserved specimen |
| 134 | 1260317975 | Peru | 1972 | Preserved specimen |
| 135 | 1260318158 | Peru | 1984 | Preserved specimen |
| 136 | 1260318234 | Peru | 1935 | Preserved specimen |
| 137 | 1260318559 | Peru | 1938 | Preserved specimen |
| 138 | 1260318690 | Peru | 1950 | Preserved specimen |
| 139 | 1260805291 | Peru | 2004 | Preserved specimen |
| 140 | 1260805340 | Peru | 2004 | Preserved specimen |
| 141 | 1261491073 | Peru | 1978 | Preserved specimen |
| 142 | 1261492791 | Peru | 1984 | Preserved specimen |
| 143 | 1261529008 | Peru | 1981 | Preserved specimen |
| 144 | 1261847020 | Peru | 1935 | Preserved specimen |
| 145 | 1262240018 | Peru | 1982 | Preserved specimen |
| 146 | 1262240869 | Peru | 1984 | Preserved specimen |
| 147 | 1262253163 | Peru | 1983 | Preserved specimen |
| 148 | 1301681369 | Peru | 1964 | Occurrence |
| 149 | 1302594862 | Peru | 1936 | Occurrence |
| 150 | 1302594878 | Peru | 1936 | Occurrence |
| 151 | 1302594910 | Peru |  | Occurrence |
| 152 | 1302594912 | Peru | 1936 | Occurrence |
| 153 | 1302594936 | Peru | 1936 | Occurrence |
| 154 | 1304894986 | Peru |  | Occurrence |
| 155 | 1320140373 | Peru |  | Preserved specimen |
| 156 | 1454506281 | Peru | 2013 | Living specimen |
| 157 | 1802484730 | Peru | 1984 | Preserved specimen |
| 158 | 1825751727 | Peru | 2013 | Preserved specimen |
| 159 | 1848789435 | Peru | 2018 | Preserved specimen |
| 160 | 1852087006 | Peru | 1938 | Preserved specimen |
| 161 | 1852093280 | Peru | 1938 | Preserved specimen |
| 162 | 1852094121 | Peru | 1938 | Preserved specimen |
| 163 | 1852096255 | Peru | 1938 | Preserved specimen |
| 164 | 1852099492 | Peru | 1938 | Preserved specimen |
| 165 | 1852100584 | Peru | 1946 | Preserved specimen |
| 166 | 1852107141 | Peru |  | Preserved specimen |
| 167 | 1852107409 | Peru |  | Preserved specimen |
| 168 | 1852112431 | Peru | 1936 | Preserved specimen |
| 169 | 1852114405 | Peru | 1939 | Preserved specimen |
| 170 | 1852115484 | Peru | 1964 | Preserved specimen |
| 171 | 1852119125 | Peru | 1949 | Preserved specimen |
| 172 | 1852119452 | Peru |  | Preserved specimen |
| 173 | 1852121369 | Peru | 1957 | Preserved specimen |
| 174 | 1852129969 | Peru | 1936 | Preserved specimen |
| 175 | 1852131949 | Peru | 1940 | Preserved specimen |
| 176 | 1852135663 | Peru | 1938 | Preserved specimen |
| 177 | 1852137157 | Peru | 1957 | Preserved specimen |
| 178 | 1852138877 | Peru | 1936 | Preserved specimen |
| 179 | 1852144424 | Peru | 1936 | Preserved specimen |
| 180 | 1852150055 | Peru | 1927 | Preserved specimen |
| 181 | 1852151776 | Peru | 1949 | Preserved specimen |
| 182 | 1852153282 | Peru | 1947 | Preserved specimen |
| 183 | 1852154687 | Peru | 1939 | Preserved specimen |
| 184 | 1852157421 | Peru | 1984 | Preserved specimen |
| 185 | 1936225460 | Peru |  | Preserved specimen |
| 186 | 2252404583 | Peru | 1948 | Preserved specimen |
| 187 | 2252407371 | Peru | 1999 | Preserved specimen |
| 188 | 2252408468 | Peru | 1948 | Preserved specimen |
| 189 | 2252410311 | Peru | 2013 | Preserved specimen |
| 190 | 2252411183 | Peru | 1971 | Preserved specimen |
| 191 | 2268888840 | Peru | 2014 | Preserved specimen |
| 192 | 2268890298 | Peru | 2014 | Preserved specimen |
| 193 | 2268890416 | Peru | 2014 | Preserved specimen |
| 194 | 2268891559 | Peru | 2014 | Preserved specimen |
| 195 | 2626924263 | Peru | 1956 | Preserved specimen |
| 196 | 2626931269 | Peru | 1957 | Preserved specimen |
| 197 | 2626949268 | Peru | 1956 | Preserved specimen |
| 198 | 2996487889 | Peru |  | Preserved specimen |
| 199 | 2996487890 | Peru |  | Preserved specimen |
| 200 | 3058979427 | Peru | 2018 | Human observation |
| 201 | 3112291311 | Peru | 2017 | Human observation |
| 202 | 3112704310 | Peru | 2017 | Human observation |
| 203 | 3327935306 | Peru | 2018 | Human observation |
| 204 | 3330696313 | Peru | 2017 | Human observation |
| 205 | 3335409306 | Peru | 2017 | Human observation |
| 206 | 3469912107 | Peru |  | Preserved specimen |
| 207 | 3497597523 | Peru | 2020 | Preserved specimen |
| 208 | 3497598516 | Peru | 2020 | Preserved specimen |
| 209 | 3497600513 | Peru | 2020 | Preserved specimen |
| 210 | 3497605517 | Peru | 2020 | Preserved specimen |
| 211 | 3902988142 | Peru | 2022 | Living specimen |
| 212 | 1897619138 | New Zealand |  | Preserved specimen |
| 213 | 1897638988 | New Zealand |  | Preserved specimen |
| 214 | 136434615 | Netherlands |  | Preserved specimen |
| 215 | 136434616 | Netherlands |  | Preserved specimen |
| 216 | 1301855965 | Netherlands |  | Occurrence |
| 217 | 1301916356 | Netherlands |  | Occurrence |
| 218 | 1302594847 | Italy |  | Occurrence |
| 219 | 136434642 | Germany |  | Preserved specimen |
| 220 | 1302108469 | Germany |  | Occurrence |
| 221 | 1302168400 | Germany |  | Occurrence |
| 222 | 1455565454 | Germany | 2012 | Preserved specimen |
| 223 | 3855095464 | Germany | 1935 | Preserved specimen |
| 224 | 3855096508 | Germany | 1934 | Preserved specimen |
| 225 | 474756493 | France | 1827 | Preserved specimen |
| 226 | 328183487 | Ecuador | 1955 | Preserved specimen |
| 227 | 328183488 | Ecuador | 1955 | Preserved specimen |
| 228 | 912044216 | Ecuador |  | Preserved specimen |
| 229 | 1057641151 | Ecuador | 2005 | Preserved specimen |
| 230 | 1096646444 | Ecuador | 1967 | Preserved specimen |
| 231 | 1096646453 | Ecuador | 1955 | Preserved specimen |
| 232 | 1096646463 | Ecuador | 1967 | Preserved specimen |
| 233 | 1096646474 | Ecuador | 1955 | Preserved specimen |
| 234 | 1096775127 | Ecuador | 1974 | Preserved specimen |
| 235 | 1258363105 | Ecuador | 1974 | Preserved specimen |
| 236 | 1258403822 | Ecuador | 1967 | Preserved specimen |
| 237 | 1258403840 | Ecuador | 1955 | Preserved specimen |
| 238 | 1258403856 | Ecuador | 1967 | Preserved specimen |
| 239 | 1258403862 | Ecuador | 1955 | Preserved specimen |
| 240 | 1258403937 | Ecuador | 1974 | Preserved specimen |
| 241 | 1258403942 | Ecuador | 1974 | Preserved specimen |
| 242 | 1258403982 | Ecuador | 1945 | Preserved specimen |
| 243 | 1258407175 | Ecuador | 1980 | Preserved specimen |
| 244 | 1258407185 | Ecuador | 1980 | Preserved specimen |
| 245 | 1258407788 | Ecuador | 1943 | Preserved specimen |
| 246 | 1260114191 | Ecuador | 1967 | Preserved specimen |
| 247 | 1262239793 | Ecuador | 1835 | Preserved specimen |
| 248 | 1852101849 | Ecuador | 1945 | Preserved specimen |
| 249 | 1852120875 | Ecuador | 1955 | Preserved specimen |
| 250 | 2515446717 | Ecuador | 1974 | Preserved specimen |
| 251 | 2517520711 | Ecuador | 1974 | Preserved specimen |
| 252 | 2517595720 | Ecuador | 1975 | Preserved specimen |
| 253 | 2618419325 | Ecuador | 1967 | Preserved specimen |
| 254 | 2618428314 | Ecuador | 1967 | Preserved specimen |
| 255 | 2618591350 | Ecuador | 1967 | Preserved specimen |
| 256 | 2626927794 | Ecuador | 1970 | Preserved specimen |
| 257 | 3456417248 | Ecuador | 2021 | Preserved specimen |
| 258 | 3855260819 | Ecuador | 1994 | Preserved specimen |
| 259 | 3855262426 | Ecuador | 1985 | Preserved specimen |
| 260 | 3855277646 | Ecuador | 1963 | Preserved specimen |
| 261 | 1844892565 | Brazil | 2017 | Preserved specimen |
| 262 | 1853943781 | Brazil | 2018 | Preserved specimen |
| 263 | 437913177 | Bolivia |  | Preserved specimen |
| 264 | 1135482942 | Bolivia | 1816 | Preserved specimen |
| 265 | 1259010425 | Bolivia | 1992 | Preserved specimen |
| 266 | 1259010441 | Bolivia | 1992 | Preserved specimen |
| 267 | 1259010651 | Bolivia | 1991 | Preserved specimen |
| 268 | 1259011043 | Bolivia | 1958 | Preserved specimen |
| 269 | 1259011653 | Bolivia | 1982 | Preserved specimen |
| 270 | 1301681429 | Bolivia | 2001 | Occurrence |
| 271 | 2513127956 | Bolivia |  | Preserved specimen |
| 272 | 2513160643 | Bolivia |  | Preserved specimen |
| 273 | 2513161097 | Bolivia |  | Preserved specimen |
| 274 | 2514603016 | Bolivia |  | Preserved specimen |
| 275 | 2514660524 | Bolivia |  | Preserved specimen |
| 276 | 2516407434 | Bolivia |  | Preserved specimen |
| 277 | 2516446423 | Bolivia |  | Preserved specimen |
| 278 | 2516600428 | Bolivia | 1947 | Preserved specimen |
| 279 | 2516610402 | Bolivia |  | Preserved specimen |
| 280 | 2517232906 | Bolivia | 1802 | Preserved specimen |
| 281 | 3020016585 | Bolivia |  | Preserved specimen |
| 282 | 1301180065 | Afghanistan |  | Occurrence |
| 283 | 1301180072 | Afghanistan |  | Occurrence |
| 284 | 1301180080 | Afghanistan |  | Occurrence |
| 285 | 1301180089 | Afghanistan |  | Occurrence |
| 286 | 1301180098 | Afghanistan |  | Occurrence |
| 287 | 1301180107 | Afghanistan |  | Occurrence |
| 288 | 1301899259 | Afghanistan |  | Occurrence |
| 289 | 1301899272 | Afghanistan |  | Occurrence |
| 290 | 1301904793 | Afghanistan |  | Occurrence |
| 291 | 1301925714 | Afghanistan |  | Occurrence |
| 292 | 1302108491 | Afghanistan |  | Occurrence |
| 293 | 1302108519 | Afghanistan |  | Occurrence |
| 294 | 1302108547 | Afghanistan |  | Occurrence |
| 295 | 1302168416 | Afghanistan |  | Occurrence |
| 296 | 1302168437 | Afghanistan |  | Occurrence |
| 297 | 1302168456 | Afghanistan |  | Occurrence |
| 298 | 1302465299 | Afghanistan |  | Occurrence |
| 299 | 1302465326 | Afghanistan |  | Occurrence |
| 300 | 1302594879 | Afghanistan |  | Occurrence |
| 301 | 1302594882 | Afghanistan |  | Occurrence |
| 302 | 1302594899 | Afghanistan |  | Occurrence |
| 303 | 1302594900 | Afghanistan |  | Occurrence |
| 304 | 1302594901 | Afghanistan |  | Occurrence |
| 305 | 1302594909 | Afghanistan |  | Occurrence |
| 306 | 1302594919 | Afghanistan |  | Occurrence |
| 307 | 1302594924 | Afghanistan |  | Occurrence |
| 308 | 1302594942 | Afghanistan |  | Occurrence |
| 309 | 1302594983 | Afghanistan |  | Occurrence |
| 310 | 41234618 |  |  | Occurrence |
| 311 | 41234670 |  |  | Occurrence |
| 312 | 41234673 |  |  | Occurrence |
| 313 | 41234675 |  |  | Occurrence |
| 314 | 41234676 |  |  | Occurrence |
| 315 | 136434610 |  |  | Preserved specimen |
| 316 | 136434611 |  |  | Preserved specimen |
| 317 | 136434612 |  |  | Preserved specimen |
| 318 | 136434633 |  |  | Preserved specimen |
| 319 | 136434634 |  |  | Preserved specimen |
| 320 | 230943015 |  |  | Preserved specimen |
| 321 | 230943027 |  |  | Preserved specimen |
| 322 | 230943028 |  |  | Preserved specimen |
| 323 | 230943029 |  |  | Preserved specimen |
| 324 | 230943030 |  |  | Preserved specimen |
| 325 | 230943031 |  |  | Preserved specimen |
| 326 | 230943035 |  |  | Preserved specimen |
| 327 | 323475537 |  |  | Living specimen |
| 328 | 328183552 |  |  | Preserved specimen |
| 329 | 328183580 |  |  | Preserved specimen |
| 330 | 474626563 |  |  | Preserved specimen |
| 331 | 474669310 |  | 1860 | Preserved specimen |
| 332 | 474669311 |  | 1860 | Preserved specimen |
| 333 | 474669312 |  |  | Preserved specimen |
| 334 | 474756494 |  |  | Preserved specimen |
| 335 | 474756495 |  |  | Preserved specimen |
| 336 | 474800349 |  | 1813 | Preserved specimen |
| 337 | 474843508 |  |  | Preserved specimen |
| 338 | 474941209 |  | 1860 | Preserved specimen |
| 339 | 685368844 |  |  | Living specimen |
| 340 | 694576418 |  |  | Preserved specimen |
| 341 | 694583187 |  |  | Preserved specimen |
| 342 | 694607459 |  |  | Preserved specimen |
| 343 | 694616947 |  |  | Preserved specimen |
| 344 | 694616948 |  |  | Preserved specimen |
| 345 | 735566583 |  |  | Living specimen |
| 346 | 931041607 |  |  | Preserved specimen |
| 347 | 931041628 |  |  | Preserved specimen |
| 348 | 1056217051 |  |  | Preserved specimen |
| 349 | 1096702912 |  |  | Preserved specimen |
| 350 | 1228024113 |  |  | Preserved specimen |
| 351 | 1228028234 |  |  | Preserved specimen |
| 352 | 1228033212 |  |  | Preserved specimen |
| 353 | 1300498039 |  |  | Occurrence |
| 354 | 1300498239 |  |  | Occurrence |
| 355 | 1300498242 |  |  | Occurrence |
| 356 | 1300498260 |  |  | Occurrence |
| 357 | 1300498264 |  |  | Occurrence |
| 358 | 1300498265 |  |  | Occurrence |
| 359 | 1302594856 |  |  | Occurrence |
| 360 | 1503355915 |  |  | Preserved specimen |
| 361 | 1703061480 |  |  | Preserved specimen |
| 362 | 1706066328 |  |  | Living specimen |
| 363 | 1717216706 |  |  | Human observation |
| 364 | 1807421066 |  |  | Preserved specimen |
| 365 | 1825736184 |  |  | Preserved specimen |
| 366 | 1848865979 |  |  | Preserved specimen |
| 367 | 1848866307 |  |  | Preserved specimen |
| 368 | 2250289171 |  |  | Material sample |
| 369 | 2611994314 |  |  | Material citation |
| 370 | 3346237714 |  |  | Preserved specimen |
| 371 | 3346482058 |  |  | Preserved specimen |
| 372 | 3346524229 |  |  | Preserved specimen |
| 373 | 3349763977 |  |  | Preserved specimen |
| 374 | 3415127136 |  |  | Preserved specimen |
| 375 | 3427778949 |  |  | Preserved specimen |
| 376 | 3427930245 |  |  | Material sample |
| 377 | 3443392154 |  |  | Human observation |
| 378 | 3817691867 |  |  | Preserved specimen |
| 379 | 3900909507 |  |  | Human observation |
| 380 | 3924369327 |  |  | Preserved specimen |

***References**

GBIF.Org (11 December 2022) GBIF Occurrence Download https://doi.org/10.15468/dl.rsh6cx.
